# The STAT3 inhibitor Stattic acts independently of STAT3 to decrease histone acetylation and modulate gene expression

**DOI:** 10.1074/jbc.RA120.016645

**Published:** 2020-12-25

**Authors:** Dipak K. Poria, Namratha Sheshadri, Kuppusamy Balamurugan, Shikha Sharan, Esta Sterneck

**Affiliations:** Laboratory of Cell and Developmental Signaling, Center for Cancer Research (CCR), National Cancer Institute (NCI), National Institutes of Health (NIH), Frederick, Maryland, USA

**Keywords:** STAT transcription factor, inhibitor, histone deacetylase (HDAC), histone acetylase (HAT), lysine acetylase (KAT), TXNRD1, acetylation, breast, prostate, cancer, DMEM, Dulbecco's modified Eagle's medium, EGFP, enhanced green fluorescent protein, FBS, fetal bovine serum, HAT, histone acetylase, HDAC, histone deacetylase, PI, propidium iodide, STAT3, signal transducer and activator of transcription 3

## Abstract

Signal transducer and activator of transcription 3 (STAT3) is an important transcription factor involved in many physiological functions including embryonic development and immune responses and is often activated under pathological conditions such as cancer. Strategies to inactivate STAT3 are being pursued as potential anticancer therapies and have led to the identification of Stattic (6-nitrobenzo[b]thiophene-1,1-dioxide) as a “specific” STAT3 inhibitor that is often used to interrogate STAT3-mediated gene expression *in vitro* and *in vivo*. Here, we show that Stattic exerts many STAT3-independent effects on cancer cells, calling for reassessment of results previously ascribed to STAT3 functions. Studies of the STAT3-deficient prostate cancer cell line PC-3 (PC3) along with STAT3-proficient breast cancer cell lines (MDA-MB-231, SUM149) revealed that Stattic attenuated histone acetylation and neutralized effects of the histone deacetylase (HDAC) inhibitor romidepsin. In PC3 cells, Stattic alone inhibited gene expression of *CCL20* and *CCL2*, but activated expression of *TNFA*, *CEBPD*, *SOX2*, and *MYC*. In addition, we found that Stattic promoted autophagy and caused cell death. These data point to profound epigenetic effects of Stattic that are independent of its function as a STAT3 inhibitor. Our results demonstrate that Stattic directly or indirectly reduces histone acetylation and suggest reevaluation of Stattic and related compounds as polypharmacological agents through multipronged cytotoxic effects on cancer cells.

Gene expression is regulated by a complex intersection of DNA-binding transcription factors and their cofactors as well as the three-dimensional genome organization, which is in part determined by protein modifications on histones. Lysine/histone acetylases (KATs/HATs) belong to the “writers” of histone codes and contribute to the opening of chromatin and activation of gene expression. Histone deacetylases (HDACs) in turn are “erasers” and remove these modifications and typically cause chromatin compaction. In the case of cancer cells, HDACs can be responsible for the silencing of tumor suppressor genes, which is one rationale for the development of many different HDAC inhibitors as potential cancer therapeutics (1). Romidepsin is one such agent and is approved for the treatment of T-ALL and in clinical trials for combination therapies in solid cancers (2).

Transcription factors that regulate gene promoters by DNA sequence-specific binding have proven difficult to target pharmacologically. Nevertheless, many agents have been explored for inhibition of STAT3, which is activated in many types of cancer (3, 4). STAT3 is a latent transcription factor, which resides in the cytoplasm as a monomer. Upon activation by upstream kinases in response to cytokines and growth factors, STAT3 is phosphorylated at Y705 within its SH2 domain, dimerizes, and translocates into the nucleus to regulate target gene expression (5). Stattic is a small molecule that emerged from a functional screen and inhibits the STAT3 SH2 domain regardless of its phosphorylation status (6). Because Stattic was initially shown to selectively inhibit STAT3, but not the related factors STAT1 and STAT5, Stattic has enjoyed popularity as a “specific” STAT3 inhibitor in mechanistic investigations of STAT3.

We and others have previously shown that STAT3 induces expression of the transcription factor C/EBPδ (encoded by the *CEBPD* gene) in response to cytokine and inflammatory signaling (7). In contrast, HDAC inhibitors, which activate the expression of many genes, suppressed *CEBPD* expression (this study, and Balamurugan *et al.*, unpublished). Our investigations into the mechanisms by which romidepsin inhibits expression of *CEBPD* led to the observation that Stattic can attenuate HDAC inhibitor-mediated *CEBPD* suppression and reduce histone acetylation independent of the presence or absence of STAT3. These findings have important implications for the interpretation of results obtained with Stattic as a STAT3 inhibitor but also raise the possibility for applications of Stattic beyond inhibition of STAT3.

## Results

### Stattic reduces histone acetylation and attenuates the effect of romidepsin on gene expression

**Figure 1:**
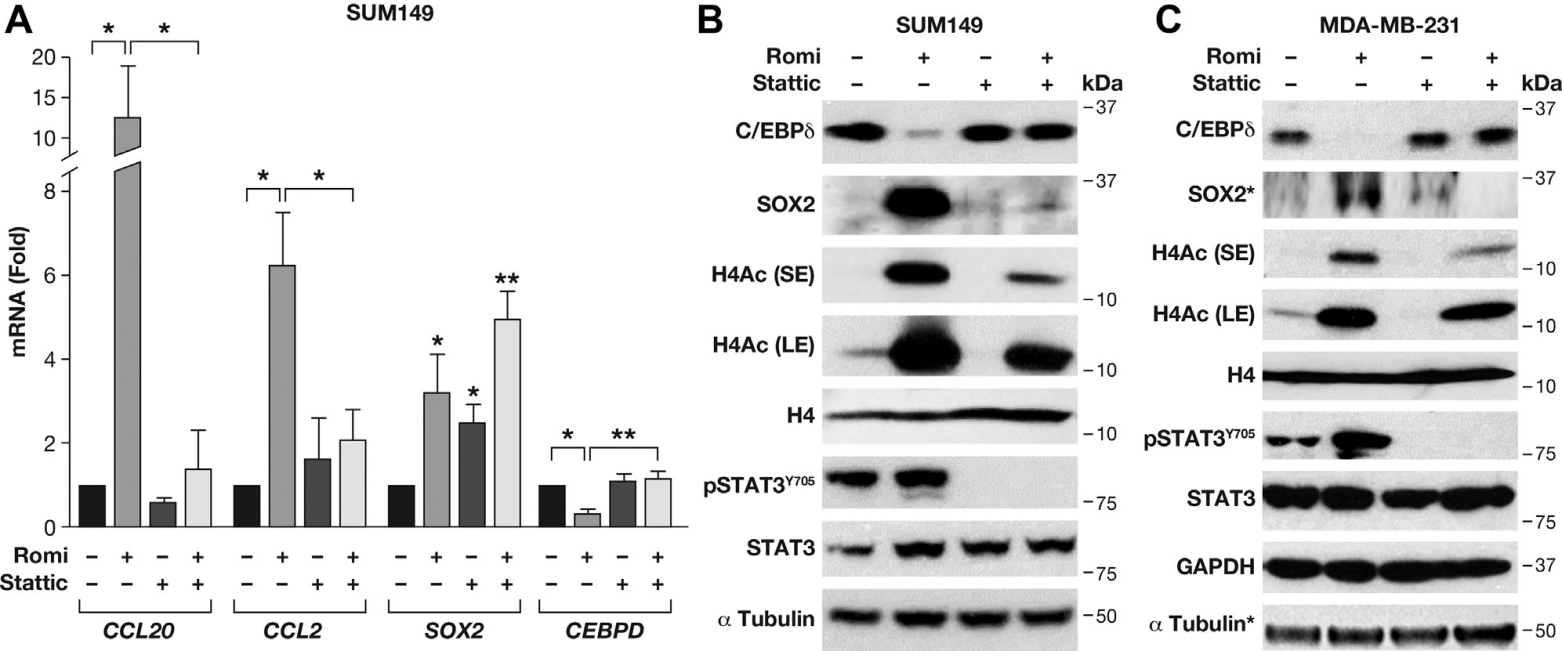
**Stattic attenuates romidepsin-mediated acetylation of histones and gene expression in human breast cancer cell lines.***A*, quantitative PCR (qRT-PCR) analysis of the indicated genes' mRNA level in SUM149 cells treated with romidepsin (Romi 10 nM) and/or Stattic (10 μM) for 8 h. Data were normalized to *GAPDH* and are represented as a fold change compared with vehicle (DMSO) treatment (mean ± SEM of three independent experiments, ∗*p* < 0.05, ∗∗*p* < 0.01). *B*, Western blot analysis of the indicated proteins and modifications in cells treated as in panel *A*. *C*, Western blot analysis as in panel *B* of MDA-MB-231 cells treated with romidepsin and/or Stattic for 8 h. SE/LE: short/long exposure. ∗Tubulin and SOX2 were from the same lysates analyzed on a separate gel.

The triple-negative inflammatory breast cancer cell line SUM149 is highly sensitive to the HDAC inhibitor romidepsin (8). Like many breast cancers, these cells also exhibit activation of the STAT3 signaling pathway (9, 10), which led us to explore the effects of romidepsin in comparison to and in combination with the STAT3 inhibitor Stattic. As expected, romidepsin increased the mRNA expression levels of several genes such as those of the pluripotency factor *SOX2* and the chemokines *CCL2* and *CCL20*. However, expression of the transcription factor *CEBPD*, which is implicated in promoting cancer cell stemness (11) was inhibited (Fig. 1*A*). Stattic alone had modest effects on the expression of *CEBPD*, *CCL2*, and *CCL20* but neutralized their response to romidepsin. Like romidepsin, Stattic induced *SOX2* expression and so did the combination treatment. At the protein level, we confirmed the respective effects of romidepsin and/or Stattic on C/EBPδ expression (Fig. 1*B*). In contrast to inducing *SOX2* mRNA, Stattic did not, however, induce SOX2 protein expression. This observation is in agreement with a prior report on Stattic inducing *SOX2* mRNA but decreasing SOX2 protein expression in MCF-7 cells (12). Similarly, Stattic prevented accumulation of SOX2 protein in romidepsin-treated SUM149 cells. With respect to acetylation, romidepsin, as expected, significantly increased polyacetylation of lysine in the amino-terminal region of histone 4 (H4Ac) and did not significantly affect STAT3 expression or phosphorylation at Y705, while Stattic completely inhibited STAT3 phosphorylation (Fig. 1*B*). Surprisingly, however, Stattic alone reduced the basal level of H4 acetylation and attenuated the accumulation of H4 acetylation caused by the HDAC inhibitor (Fig. 1*B*). To assess an alternate triple-negative breast cancer cell line, we analyzed the combination of romidepsin and Stattic in MDA-MB-231 cells (Fig. 1*C*) and observed similar effects on C/EBPδ protein expression and H4 acetylation. Taken together, these data show that Stattic has a profound effect on histone H4 acetylation and counters several of romidepsin's effects on gene and protein expression. Thus, we hypothesized that Stattic can regulate gene expression by epigenic mechanisms that involve inhibition of histone acetylation.

### Stattic reduces histone acetylation and modulates gene expression independent of STAT3 activity

**Figure 2:**
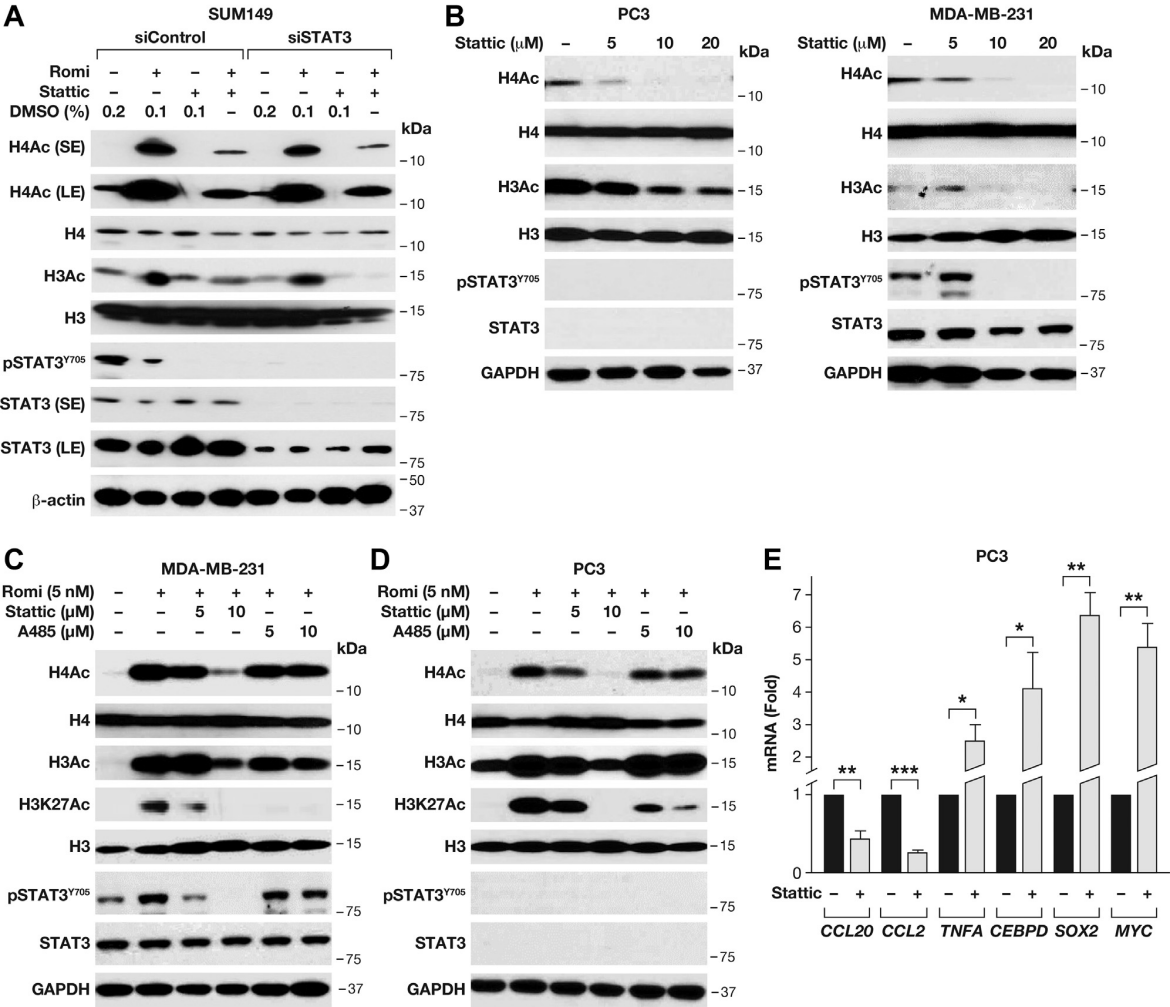
**Stattic inhibits histone acetylation and modulates gene expression independent of STAT3.***A*, Western blot analysis of the indicated proteins and modifications in SUM149 cells transfected with siRNA and treated with romidepsin (10 nM) and/or Stattic (10 μM) for 8 h, as indicated. *B*, Western blot analysis of the indicated proteins and modifications in PC3 and MDA-MB-231 cells treated with increasing doses of Stattic for 8 h. *C*, *D*, Western blot analysis of MDA-MB-231 (*C*) and PC3 (*D*) cells treated with romidepsin (5 nM) alone or in combination with the indicated doses of Stattic or A485 for 8 h. *E*, qRT-PCR analysis of the indicated genes' mRNA levels in PC3 cells treated with 10 μM Stattic for 6 h. mRNA levels were normalized to RPLP0 mRNA and are represented as fold change compared with vehicle (DMSO) treatment (mean ± SEM of three independent experiments, ∗*p* < 0.05, ∗∗*p* < 0.01, ∗∗∗*p* < 0.001). LE, long exposure; SE, short exposure.

To assess if Stattic's effect on histone acetylation was indirectly mediated by inhibition of STAT3, we first silenced STAT3 in SUM149 cells, which had, however, no effect on romidepsin-mediated H4 or H3 acetylation (Fig. 2*A*). In addition, Stattic similarly reduced basal and romidepsin-induced acetylation of H4 and/or H3 in siControl and siSTAT3 transfected cells. Because siSTAT3 had no measurable effect on the regulation of acetylation, although STAT3 was significantly depleted, off-target effects are not of concern in this experiment (Fig. 2*A*). However, because transient silencing of gene expression rarely eliminates its target completely, we next compared genetically STAT3-deficient PC3 prostate cancer cells and the STAT3-proficient MDA-MB-231 breast cancer cells. In both cell lines, we observed similar dose-dependent inhibition of histone H3 and H4 acetylation (Fig. 2*B*). STAT3 expression and inhibition of STAT3 phosphorylation by Stattic could only be observed in MDA-MB-231 cells as STAT3 is deleted in PC3 cells (13, 14). When combined with romidepsin, Stattic could avert the HDAC inhibitor-induced hyperacetylation of H4 and H3 as well as H3K27 acetylation (Fig. 2, *C–D*). As expected, Stattic inhibited STAT3 phosphorylation in MDA-MB-231 cells also in the presence of romidepsin. Efficient inhibition of STAT3 was seen with 10 μM of Stattic, which is the concentration that has been used in many studies for STAT3 inhibition. At this dose, Stattic significantly attenuated basal acetylation as well as hyperacetylation of histones by romidepsin even in STAT3-deficient cells. These data raised the hypothesis that Stattic may directly or indirectly inhibit histone acetylation but independent of its effect on STAT3. Treatment with A485, an inhibitor of the CBP/p300 family of HATs, significantly reduced specifically acetylation of its target H3K27 (15) in both romidepsin-treated cell lines but only modestly reduced H3 and H4 polyacetylation levels (Fig. 2, *C–D*). These data suggest that Stattic—if acting as a HAT inhibitor—is not selective for the CBP/p300 family.

Changes in epigenetic marks on chromatin, such as histone acetylation, alter gene expression. Thus, we assessed the effect of Stattic on the expression levels of several other known STAT3 target genes such as the chemokines CCL20 and CCL2 (16) and TNFα (17) in PC3 cells. Stattic treatment inhibited gene expression of *CCL20* and *CCL2* but induced mRNA expression of *TNFA*, *CEBPD*, *SOX2*, as well as *MYC* in PC3 cells (Fig. 2*E*). Collectively, these data demonstrate that Stattic decreases histone modification by acetylation similarly in the presence and absence of STAT3 and alters gene expression patterns independent of its activity as STAT3 inhibitor.

### Stattic reduces cell survival and promotes autophagy independent of STAT3 inhibition

**Figure 3:**
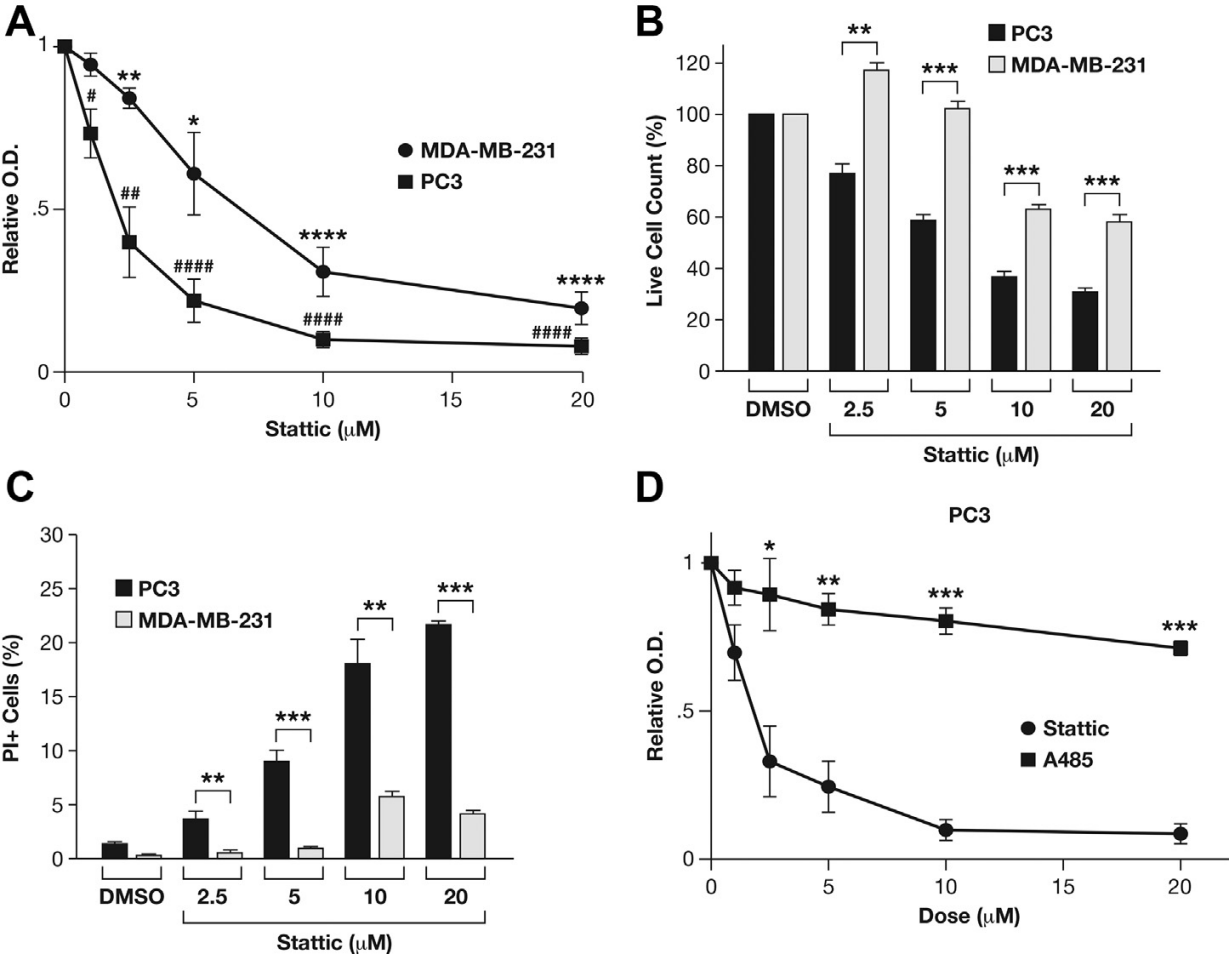
**Stattic induces cell death in STAT3-null cells.***A*, MTT analysis of relative cell viability of MDA-MB-231 and PC3 cells treated with increasing doses of Stattic for 24 h (mean ± SEM of three independent experiments, ∗*p* < 0.05, ∗∗*p* < 0.01, ∗∗∗*p* < 0.001, ∗∗∗∗*p* < 0.0001). *B*, *C*, image cytometric quantification of live (*B*) and dead/dying (*C*) cells treated as in panel *A* and in the presence of propidium iodide (mean ± SEM of three independent experiments, ∗*p* < 0.05, ∗∗*p* < 0.01, ∗∗∗*p* < 0.001 compared with vehicle control for each cell line). *D*, relative cell viability measured by MTT assay of PC3 cells treated with increasing doses of Stattic or A485 for 24 h (mean ± SEM of three independent experiments, ∗*p* < 0.05, ∗∗*p* < 0.01, ∗∗∗*p* < 0.001, comparison of treatments at each time point).

STAT3 promotes cell proliferation and survival in many types of cancer cells. Thus, we compared the effect of Stattic on cell survival/proliferation in MDA-MB-231 and PC3 cells by MTT assay (Fig. 3*A*). As expected (6), Stattic exerted dose-dependent cytotoxicity on MDA-MB-231 cells. However, STAT3-deficient PC3 cells were even more sensitive to Stattic treatment with an EC50 of 1.7 μM compared with 5.5 μM for MDA-MB-231 cells. Quantification of live and dead/dying cells based on exclusion of propidium iodide confirmed that PC3 cells were more sensitive than MDA-MB-231 cells (Fig. 3, *B–C*), while also showing that the metabolic MTT assay overestimated the cytotoxicity of Stattic. In comparison, the CBP/p300 inhibitor A485 was relatively well tolerated by PC3 cells (Fig. 3*D*). These results demonstrate that the cytotoxicity of Stattic can be due to not only inhibition of STAT3 but also STAT3 -independent effects, which may be related to interference with histone acetylation.

**Figure 4:**
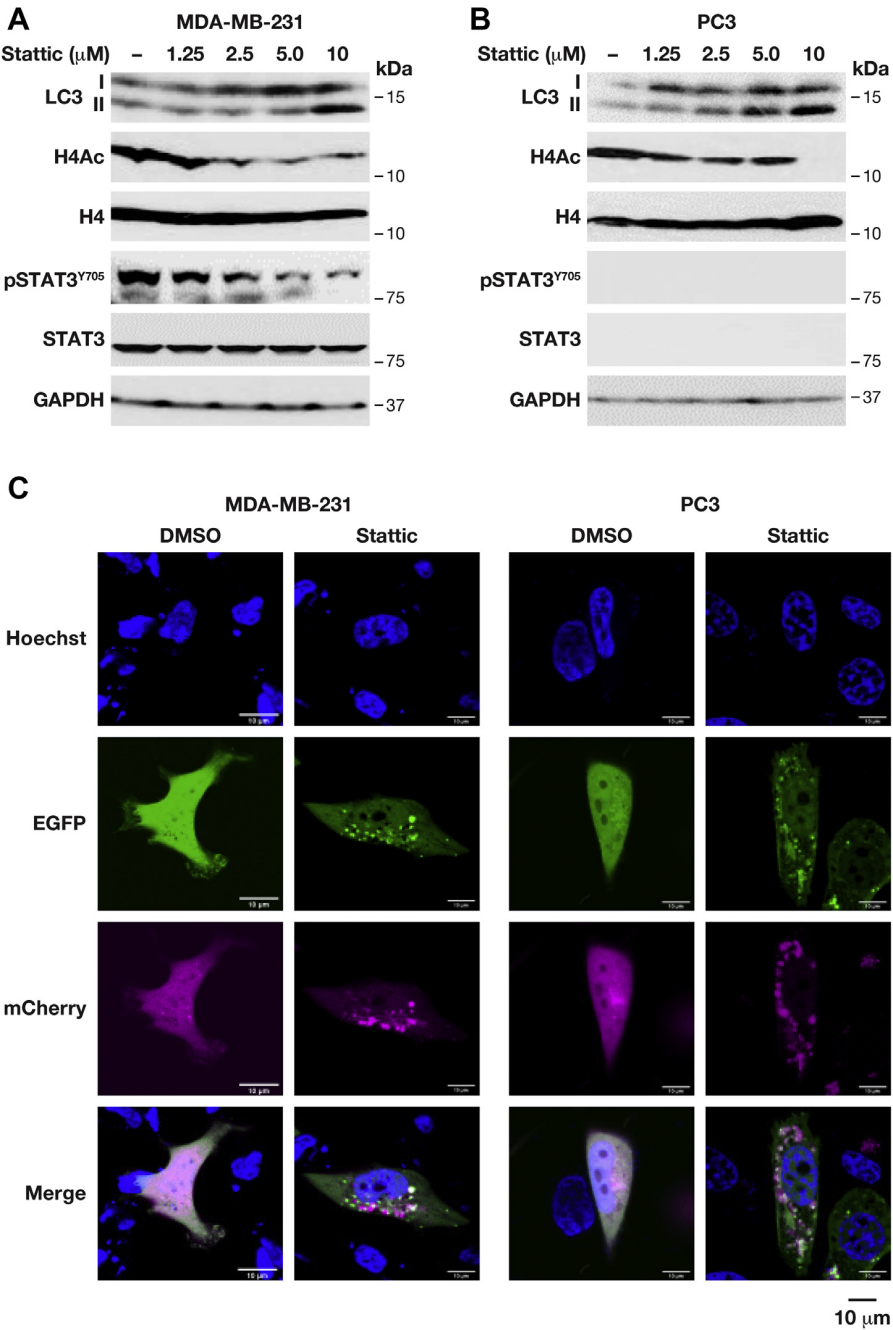
**Stattic induces autophagy in STAT3-null cells.***A*, *B*, Western blot analysis of the indicated proteins and modifications in MDA-MB-231 (*A*) and PC3 cells (*B*) treated with increasing doses of Stattic for 24 h. *C*, representative fluorescent microscopy images of MDA-MB-231 and PC3 cells transiently transfected with mCherry-EGFP-LC3B reporter, followed by treatment with vehicle (DMSO) or Stattic for 8 h. Hoechst dye was added prior to image acquisition to visualize nuclei. EGFP, enhanced green fluorescent protein.

STAT3 has been shown to repress autophagy and consequently several STAT3 inhibitors including Stattic promote autophagy (18). Autophagy can be assessed by the ratio of LC3-I and LC3-II isoforms with the latter representing the lipidated form that is required for fusion of autophagosomes with lysosomes (19). Treatment with Stattic increased LC3 expression and lipidation (LC3-II) in MDA-MB-231 as well as PC3 cells along with inhibition of H4Ac as described above (Fig. 4, *A–B*). Accumulation of LC3-II can be due to increased autophagy or inhibition of the fusion of autophagosomes with lysosomes (19). As an alternative approach to assess autophagy, we expressed an LC3B reporter fused with enhanced green fluorescent protein (EGFP) and mCherry, which marks autophagosomes yellow and autophago-lysosomes red due to interference of the acidic environment with EGFP fluorescence (19). Upon treatment with Stattic for 8 h, the LC3B fusion protein became localized to puncta that were either yellow or red (Fig. 4*C*). This result shows that Stattic increased autophagic vesicle formation in MDA-MB-231, as reported previously (18). However, the same response was observed in STAT3-null PC3 cells, indicating that STAT3 is not necessary for the induction of autophagic vesicle formation in these cells. Taken together, analysis of cytotoxicity and autophagy indicates that several of the cellular effects of Stattic that have previously been attributed to inhibition of STAT3 can occur similarly in STAT3-deficient cells.

**Figure 5:**
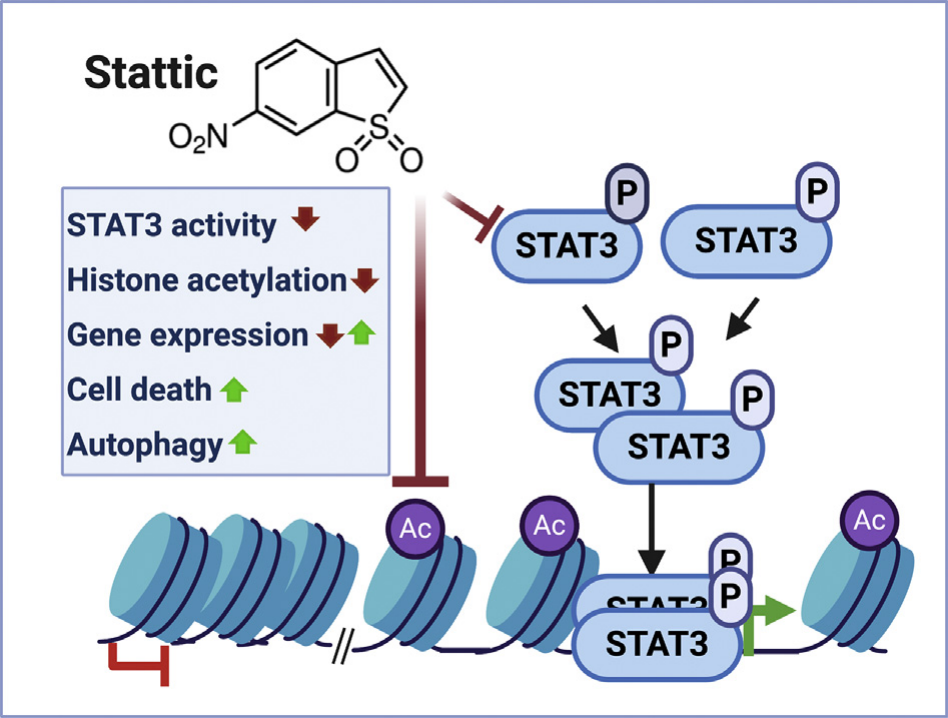
**Model illustrating that Stattic can affect gene expression, autophagy, and survival through STAT3-dependent and -independent mechanisms.** Not depicted, these two mechanisms may result in complex intersections in cells with activated STAT3.

## Discussion

The quest for pharmacological inhibitors of oncogenic proteins and signaling pathways has yielded a plethora of compounds as research tools. However, few compounds are highly specific and/or become useful clinical therapeutics. Thus, similar to other STAT3 inhibitors, Stattic is expected to exert nonspecific effects (3, 20). Although initially proclaimed as specific for STAT3, Stattic was later shown to also inhibit phosphorylation of other STAT family members (20, 21). Possibly, the discrepancy in results could be due to differences between cell types and also raise the possibility for indirect mechanisms of inhibition. Indeed, while Stattic was shown to directly alkylate STAT3 (22), indirect inactivation of STAT3 *via* irreversible inhibition of the oxidoreductase TrxR1 (*TXNRD1*) was reported as a mechanism of several STAT3 inhibitors including Stattic (23). Our study revealed that Stattic treatment reduces the levels of histone acetylation independent of STAT3 activity.

While HAT and HDAC protein families were named based on histones as prominent targets, many if not all cellular proteins can be modified by lysine acetylation, and the effects of inhibitors of either family will have profound effects on protein functions beyond genome organization. Intriguingly, TrxR1 is activated through acetylation (24), suggesting that Stattic inhibition of TrxR1 could be due to inhibition of its acetylation. STAT3 itself is a target of acetylation including by the HAT CBP/p300, which promotes STAT3 dimerization and activity without affecting its phosphorylation (25, 26, 27, 28). On the other hand, HDACs are involved in termination of STAT3 activation (25), which may explain the accumulation of pSTAT3^Y705^ occasionally observed in our experiments after romidepsin treatment (*e.g.*, Figs. 1*C* and 2*C*). In light of the reduction of histone acetylation by Stattic, the potential effect of Stattic on reducing STAT3 acetylation cannot be ruled out as part of its mechanism as a STAT3 inhibitor.

Our results could lead to the speculation that Stattic inhibits histone acetylation. There has been considerable interest in targeting epigenetic regulators in cancer and other diseases. However, while several HDAC inhibitors are currently used in the clinic, development of HAT inhibitors has proven significantly more challenging (29, 30). Many HAT inhibitors were shown to be unspecific thiol-reactive compounds (31). Such concerns do not, however, negate their activity as inhibitors of histone acetylation. In turn, Stattic may also be classified as a “thiol-reactive” compound, and it is therefore conceivable that Stattic's effect on histone acetylation is mechanistically similar to that of compounds that were initially identified or characterized as HAT inhibitors. Conversely, curcumin is a natural compound that can inhibit p300/CBP besides other enzymes (29) and inhibits STAT3 through interaction with cysteine (32). Reducing agents such as DTT and N-acetylcysteine neutralize the STAT3 inhibitory function of Stattic as well as that of thiol-reactive HAT inhibitors (31, 32). Interestingly, though, N-acetylcysteine's ability to counteract Stattic may not always depend on its antioxidant property (33). Lastly, because histone acetylation depends also on the availability of acetyl-CoA, compounds that perturb metabolism may similarly result in reduced histone acetylation. To conclude, while the mechanism by which Stattic reduces histone acetylation remains to be determined, we have demonstrated that it does so at the same concentrations at which STAT3 is efficiently inhibited. This activity is independent of STAT3 and, therefore, needs to be considered in the interpretation of results obtained with this drug as indicated in Figure 5. As it was done in many but not all reports on functional studies of STAT3, results obtained with Stattic as a STAT3 inhibitor will need to be validated with alternative approaches such as RNA interference or genetic deletion of STAT3.

Lastly, advanced prostate cancer, including the PC3 line used here, has comparatively low HAT activity (34). This may be a reason for the high sensitivity of PC3 to inhibition of histone acetylation. Despite the controversies surrounding HAT inhibitors, our data suggest that this pathway could be further explored as potentially targetable in advanced prostate cancer.

## Experimental procedures

### Cell culture and treatments

MDA-MB-231 cells were obtained from ATCC. The PC3 cell line originated from the NCI Cell Line Repository and SUM149 cells from Asterand Bioscience. Cell lines were last authenticated in 2020 (PC3) and 2017 (SUM149, MDA-MB-231). Cells were cultured in a 5% CO_2_ incubator at 37 °C and ambient oxygen levels. MDA-MB-231 cells were cultured in Dulbecco's modified Eagle's medium (DMEM), PC3 cells in RPMI-1640 media, and SUM149 cells in Ham's F-12 media with 5 μg/ml hydrocortisone and 1 μg/ml Insulin. All media were supplemented with 10% fetal bovine serum (FBS), 100 units/ml penicillin, and 100 μg/ml streptomycin. The SUM149 data in Figure 1 were obtained from suspension sphere cultures in human MammoCult medium (Stem Cell Tech #05621) supplemented with 1% proliferation supplement (Stem Cell Tech #05622) in ultralow attachment culture dishes for 3 days before drug treatments. Cells were treated with indicated doses of Stattic, romidepsin, or A485 for indicated times in the corresponding culture media. DMSO was used as vehicle control in all the treatments. Reagents used in cell culture were from Selleck Chemicals (Stattic, #S7014; romidepsin, #S3020) Thermo Fisher Scientific (MTT, #M-6494; DPBS, #14190-144; DMEM, #11965-092; F12/GlutaMax media, #31765-035, Pen/Strep, #15140122; L-Glutamine, #25030081), MilliporeSigma (Hydrocortisone, #H4001; Insulin, #10516; DMSO, #D-2650; Propidium Iodide, #P4170), and Quality Biologicals (RPMI-1640, #112-024-101).

### Cell viability/death assay

To assess cell viability by MTT (3-(4,5-dimethylthiazolyl-2)-2,5-diphenyltetrazolium bromide) assay, cells were seeded in 96-well plates and the next day triplicate wells were treated with different doses of Stattic, A485, or vehicle for 24 h followed by incubation with 5 mg/ml MTT reagent for 30 min. Formazan crystals formed inside the cells were lysed and dissolved by DMSO. Absorption was measured at 570 nm using a plate reader and represented as percent of DMSO control. Total cell counts and propidium iodide (PI) positive cell counts were assessed with Celigo imaging cytometer (Nexcelom Biosciences) after staining with 0.5 ug/ml propidium iodide (PI). Live cells (PI negative) are presented as % of DMSO control, and dead cells (PI positive) are presented as percent of the total cell number.

### Transient siRNA transfection

SUM149 cells were nucleofected with siRNA (pool of three siSTAT3 sequences, Santa Cruz Biotechnology Inc, sc-29493; siControl, 5′-CGUACGCGGAAUACUUCGAUUdTdT-3′) using AMAXA nucleofector program #O-005. Two days later, cells were passaged, and the following day treated as indicated.

### Cell lysis and Western blotting

Whole-cell extracts were prepared by lysis with 2X Laemmli sample buffer (BioRad, Cat# 1610737) followed by heating at 100 °C for 5 min. Protein concentrations were measured by Pierce 660 nm protein assay reagent (Thermo Fisher, cat#1861426) in the presence of IDCR reagent (Thermo Fisher, cat#22663) according to manufacturer's protocol. About 20 μg of proteins was loaded onto 4–20% Tris-glycine polyacrylamide gels and Western analyses were carried out using standard procedures. Antibodies were obtained from Cell Signaling Technology (pSTAT3^Y705^, #9145; STAT3, #4904; SOX2, #3579; Acetyl-Histone H3 (K27), #8173; Histone H3, #3638; Histone H4, #2935; Anti-mouse IgG, HRP linked, #7076; Anti-Rabbit IgG, HRP linked, #7074), MilliporeSigma (Acetyl-Histone H3, #06-599; Acetyl-Histone H4, #06-866), Santa Cruz Biotechnology (CEBPD, #sc-135733; GAPDH, #sc-47724), MBL International Corp (LC3, #PM036), DSHB (α−tubulin, #12G10), and Abcam (Actin, #ab6276).

### RNA isolation and quantitative PCR

Total RNA was purified by GeneJET RNA purification kit (Thermo Scientific, #K0732), and cDNA was synthesized using Superscript Reverse Transcriptase III (RT) according to manufacturer's instructions (Invitrogen, #18080044). PCR was carried out with Fast SYBR Green master mix (#4385612, Applied Biosystems) using the QuantStudio 5 (Applied Biosystems) or 7500 Fast (Applied Biosystems) Real-Time PCR instrument. Relative expression levels were measured using ΔΔ*C*t method. Gene-specific primers were designed as follows for “Gene Name – Forward primer (5′-3′) – Reverse primer (5′-3′)”: *CEBPD* – CTGTCGGCTGAGAACGAGAA – TGAGGTATGGGTCGTTGCTG; *CCL20* – CGAATCAGAAGCAGCAAGCAA – TTGCGCACACAGACAACTTT; *CCL2* – CCGAGAGGCTGAGACTAACC – GGGGCATTGATTGCATCTGG; *TNFA* – TGTTGTAGCAAACCCTCAAGC – CTTGGTCTGGTAGGAGACGG; *SOX2* – CCATCCACACTCACGCAAAA – TATACAAGGTCCATTCCCCCG; *MYC* – AAACACAAACTTGAACAGCTA – ATTTGAGGCAGTTTACATTATG; *GAPDH* - AAGGTCGGAGTCAACGGATTTG – CCATGGGTGGAATCATATTGGAA; *RPLP0* – GCAATGTTGCCAGTGTCTGTC – GCCTTGACCTTTTCAGCAAGT.

### mCherry-EGFP-LC3B reporter assay

Autophagic vesicle formation was visualized by the mCherry-EGFP-LC3B reporter, which was a kind gift from Dr. Wei-Xing Zong (Rutgers University, School of Pharmacy). The plasmid was transiently transfected into cells. About 40 h post transfections, the cells were treated with 10 μM Stattic or vehicle control for 8 h followed by live cell imaging of the EGFP and mCherry fluorescence using an LSM710 confocal microscope. NucBlue live cell stain (Invitrogen #R37605) was used for nuclear staining.

### Statistical analysis

Unpaired *t*-test was performed to analyze data from biological replicates using GraphPad Prism software. *p* values lesser than 0.05 were considered as statistically significant.

## Data availability

All data are contained within the article.

## Conflict of interest

The authors declare that they have no conflicts of interest with the contents of this article.
